# Community Gardening Increases Vegetable Intake and Seasonal Eating From Baseline to Harvest: Results from a Mixed Methods Randomized Controlled Trial

**DOI:** 10.1016/j.cdnut.2023.100077

**Published:** 2023-04-15

**Authors:** Katherine Alaimo, Alyssa W. Beavers, Eva Coringrato, Kristin Lacy, Wenjuan Ma, Thomas G. Hurley, James R. Hébert

**Affiliations:** 1 Department of Food Science and Human Nutrition, Michigan State University, East Lansing, MI, USA; 2 Department of Nutrition and Food Science, Wayne State University, Detroit, MI, United States; 3 Center for Statistical Training and Consulting, Michigan State University, East Lansing, MI, United States; 4 Department of Epidemiology and Biostatistics, Arnold School of Public Health, University of South Carolina, Columbia, SC, United States

**Keywords:** gardening, community gardening, fruit and vegetable intake, seasonal eating

## Abstract

**Background:**

Gardening has been associated with greater fruit and vegetable intake, but few randomized trials have been conducted.

**Objectives:**

We sought: *1*) to determine changes in fruits and vegetable intake combined and separately from baseline (spring) to harvest time (fall), as well as from baseline to winter follow-up, and *2*) to identify the mediators (both quantitatively and qualitatively) between gardening and vegetable intake.

**Methods:**

A randomized controlled trial of community gardening was conducted in Denver, Colorado, USA. Post hoc quantitative difference score analysis and mediation analysis were conducted by comparing intervention group participants who were randomized to receive a community garden plot, plants and seeds, and a gardening class with control group participants who were randomized to remain on a waitlist for a community garden plot (*n* = 243). Qualitative interviews were completed with a subset of participants (*n* = 34) and analyzed to explore the influences of gardening on diets.

**Results:**

The average age of participants was 41 y, 82% of them were female, and 34% of them were Hispanic. Compared with control participants, from baseline to harvest, community gardeners significantly increased their intake of total vegetables by 0.63 servings (*P* = 0.047) and garden vegetables by 0.67 servings (*P* = 0.02) but not combined fruit/vegetable or fruit intake. There were no differences between the groups from baseline to winter follow-up. Community gardening was positively associated with eating seasonally (*P* = 0.02), which had a significant indirect effect on the association between community gardening and garden vegetable intake (bootstrap 95% CI: 0.002, 0.284). Reasons qualitative participants gave for eating garden vegetables and making dietary changes included the availability of garden produce; emotional attachment with the plants; feelings of pride, accomplishment, and self-reliance; taste and quality of garden produce; trying new foods; cooking and sharing food; and increased seasonal eating.

**Conclusions:**

Community gardening increased vegetable intake through increased seasonal eating. Community gardening should be recognized as an important setting for improving diets.

This trial was registered in ClinicalTrials.gov as NCT03089177 (https://clinicaltrials.gov/ct2/show/NCT03089177).

## Introduction

Production of fruits and vegetables through urban agriculture and community gardening has the potential to increase the sustainability and food sovereignty of local regions as well as improve the nutrient density of diets. Recent systematic reviews by Garcia et al. [1] and Hume et al. [2] found consistent evidence for cross-sectional associations between community gardening and increased fruit and vegetable intake; however, both concluded that improved methodological rigor is needed. A third review by Burt et al. found “neutral” evidence demonstrating that community gardening improves fruit and vegetable intake. In a recent national study, home garden access was associated with a 30% greater likelihood of acquiring vegetables required to meet vegetable recommendations as compared to respondents without access to a garden [3]. In another recent study, household food procurement including gardening was significantly associated with the increased intake of fruits and vegetables (measured separately), but only for food-secure households [4]. Qualitative studies of gardening and diet, nutrition, and food security have found increased access to and intake of produce among gardeners [5,6].

The Community Activation for Prevention Study (CAPS) randomized controlled trial (RCT), conducted from 2017 to 2020, was designed to determine whether community gardening improved health outcomes and health behavior, including fruit and vegetable intake, among age- and ethnically diverse healthy adults who had not been gardening in the 2 y prior to enrollment [7]. Recently, we reported the results of the study’s planned analysis, which included a time-by-treatment analysis, testing the overall differences between the control and gardening groups over time. We found that there was a significant difference in fiber intake between the gardening and control groups, but not combined fruit and vegetable intake [8]. The purpose of the present post hoc analyses of the CAPS trial was to delve more deeply into those findings with qualitative narratives from a representative subset of participants and to understand the quantitative fruit and vegetable outcomes in more detail, i.e., considering them separately rather than aggregated into 1 category, assessing the change in intake over time, and determining the mediators of effect, if any. Mixed methods studies can add important context and meaning to quantitative findings by enabling participants to describe their experiences in their own words [9].

## Methods

CAPS, an RCT designed to assess health outcomes of community gardening, was conducted in Denver and Aurora, Colorado, USA, between 2017 and 2020 in partnership with Denver Urban Gardens (DUG) (https://dug.org/), a nonprofit organization that supports community and school gardens [7]. Community gardens in the DUG network are administered by volunteer garden leaders and consist of separate garden plots available to individuals/families. A total of 291 participants were enrolled in the study in 3 waves. Randomization to 37 DUG community gardens was conducted within each community garden waitlist. Details about the trial design, protocol, and recruitment are described elsewhere [6]. The trial was registered as NCT03089177 at ClinicalTrials.gov. The study protocol was approved by the University of Colorado Boulder Institutional Review Board (16-0644) and Michigan State University Institutional Review Board (LEGACY16-1296R), and an advisory committee of DUG staff, community garden leaders, and gardeners and past American Community Gardening Association executive members helped inform the study. All study participants provided written informed consent before randomization. Study staff, investigators, and statisticians were blinded to participant randomization status until the completion of data collection and analysis of primary outcomes.

### Qualitative interviews

Qualitative interview participants were selected by purposive sampling to ensure variation in demographics, assigned community garden, and the level of gardening engagement, and were interviewed after their participation in the trial was completed. A subset of 54 intervention and control participants were contacted by phone to participate in interviews; 25 intervention participants and 24 control participants who gardened in 24 different DUG gardens agreed to be interviewed; 6 declined or did not respond to researchers. The second and fourth authors, along with 2 additional interviewers (all female), whose credentials ranged from BS to MS, conducted the 60–90 min interviews at the participants’ homes or gardens. Interviewers were research assistants for the study, interested in gardening, and were trained by qualitative methods coursework and/or by the first author. For this analysis, we included 45 interviews with 34 participants, including 23 intervention participants interviewed after their first gardening season and 11 control participants interviewed twice – prior to their first garden season and after their first gardening season. One intervention participant interview was excluded from the analysis because of incomplete data, and 12 control participant interviews were excluded because they were interviewed only before they began gardening, but not after. All participants provided a separate written informed consent prior to the qualitative interviews.

Interview guides were created for *1*) control participants prior to their first season gardening and *2*) intervention and control participants after their first season gardening. The advisory committee suggested topics for interviews and provided feedback on question wording.

Four community gardeners in Lansing, Michigan, piloted the draft interview questions to assess the suitability and ease of responding, and questions were modified accordingly. Semistructured qualitative interview topics included motivations to garden, previous gardening experiences, typical visits to the garden, social interactions with friends, family and other gardeners, perceived value of gardening, likes and dislikes about gardening, preintervention diet, and changes in diet since gardening. First and second interviews were conducted in spring 2018, winter 2018/2019, and spring 2019. Participants were provided with a $20 gift card for participating in the interviews. Interviews were tape-recorded and transcribed, and these transcripts were checked and corrected for accuracy. Three interviews were conducted in Spanish, and the transcripts were translated.

### Qualitative data analysis

Transcripts were analyzed by 5 experienced researchers (including the first through fourth authors) with Atlas.ti software using grounded theory (whereby codes and themes were constructed inductively without a pre-existing coding frame from the data) and comparative case study analysis (whereby each participants’ garden experience was analyzed as a discrete unit and compared and contrasted with the experiences of the other participants) [[10], [11], [12], [13]]. In the first stage (initial coding), researchers coded a small subset of interviews independently to generate the initial codebook based on the topics of the interviews. These interviews were then coded collaboratively by the researchers to reach consensus on code meaning and definitions. Meetings were held regularly to add codes as needed, refine and finalize the codebook, and generate notes for each interview based on a standard template. The researchers then coded the remaining interviews using the established codebook. The coding for each transcript was checked by an alternative researcher to ensure that the finalized codes were applied uniformly. Next, through regular discussion to generate themes and attention to each participant as a separate case study, standardized PowerPoint displays were created to assist with “telling the story” of each participant and enable the researchers to draw and explain connections between themes (data display). Research team discussions were employed to clarify discrepancies and review PowerPoint displays to ensure that summary statements were being written uniformly. In the final stage of analysis (results generation), summary statements for each theme were added to an excel table, and similar and disparate experiences for each participant were compared. Final summary results were written iteratively and by consensus by the first 4 authors. The findings were presented to the CAPS Advisory Committee for feedback. The codebook is available upon request.

### Dietary recalls and health questionnaires

All trial participants completed a demographic and health survey and 3 telephone-administered 24-h dietary recall interviews by experienced bilingual (English/Spanish) registered dietitians at each of the 3 data collection time points – before beginning gardening (spring), at harvest time (fall), and after harvest (winter). Interviews were conducted on randomly selected days (for the 3-d sampling scheme, 2 weekdays and 1 weekend day) during a 2-wk sampling frame. The Nutrient Data System for Research (NDS-R, version 2020; Nutrition Coordinating Center, University of Minnesota) software was used to conduct the dietary interviews and calculate dietary intake outcomes. The recalls determined to be unreliable by the interviewers were excluded from the analyses. Fruits and vegetables were categorized according to the NDS-R food groups rather than botanically, i.e., cucumbers and tomatoes were considered vegetables, not fruits. Estimates of fruit and vegetable intake excluded juice, avocados, French fries, and fried onion rings.

### Quantitative data analysis

#### Outcome variables

Outcome variables included consumption of fruits and vegetables combined and separately, and “garden vegetables,” which were vegetables that were commonly grown by the CAPS participants and included these categories: dark green vegetables, deep-yellow vegetables, tomatoes, other starchy vegetables (such as peas and corn), and other vegetables (such as carrots, cauliflower, and cucumbers), and excluded potatoes and legumes. Only 24% of the CAPS participants reported growing fruits, whereas more than 97% grew vegetables. Few participants reported growing potatoes or legumes.

#### Mediation variables

After the qualitative analysis was completed, we used the qualitative analysis results to select available variables from the health survey to be included in the mediation analysis to determine their relationships with gardening and the outcomes of interest. All variables selected were measured using validated items from the NCI Food Attitudes and Behaviors Survey. The change from baseline to harvest for the following variables was included in the mediation models: social support (3-item scale; Likert scale, agreement with statements, e.g., “my family or friends encourage me to eat fruits and vegetables”) and self-efficacy (6-item scale; Likert scale, e.g., “how confident are you that you could eat health foods, like fruits and vegetables, when you are tired?”) for fruit and vegetable intakes, sharing food with others (5-item scale; Likert scale, e.g., “how often do you share new foods with your family, friends, and neighbors?”), seasonal eating measured by a single question “I tend to eat different types of fruits and vegetables depending on what is in season”, and preferences for vegetables measured by asking participants to rate their liking of 20 vegetables [10].

### Difference score models

All difference score analyses were conducted under intent-to-treat and analyzed as randomized using R software [14]. Participants were included in the post hoc analyses if they had data at both time points analyzed except for the last data collected at the end of the study during the COVID-19 pandemic; the study investigation team met and by consensus decided to exclude these data (*n* = 88) from the analysis while being blinded to the study groups. Separate general linear mixed-effect models were fit for 2 difference scores – baseline to harvest (T1 to T2) and harvest to winter follow-up (T1 to T3) – to examine whether the difference scores in the 4 outcome variables (combined fruits and vegetables, fruit alone, vegetables alone, and garden vegetables only) differed by group assignment (alpha = 0.05 level, 2-tailed). As a first step, we used mixed-effect models to account for the autocorrelations among participants who were randomized to the same community garden. However, if the garden level did not account for the variance of the dependent variable (singularity issue), then the mixed-effect model was not appropriate and a single-level linear regression model was used instead.

### Mediation model

We used mediation modeling to explore the mechanisms that influenced participants’ change in garden vegetable intake from T1 to T2. Both direct and indirect effects of the intervention were estimated using MPlus software [15]. Indirect effects were estimated using the bootstrap method (*n* = 5000). The model was specified as follows:

(1) $${Social\;support=\alpha }_{0}^{1}+\alpha_{1}^{1}intervention+\varepsilon^{1}$$

(2) $${Share\;food=\alpha }_{0}^{2}+\alpha_{1}^{2}intervention+\varepsilon^{2}$$

(3) $${Eat\;seasonally=\alpha }_{0}^{3}+\alpha_{1}^{3}intervention+\varepsilon^{3}$$

(4) $${Try\;new\;food=\alpha }_{0}^{4}+\alpha_{1}^{4}intervention+\varepsilon^{4}$$

(5) $${Self-efficacy=\alpha }_{0}^{5}+\alpha_{1}^{5}intervention+\varepsilon^{5}$$

(6) $$Garden\;vegetable\;consumption=\beta_{0}+\beta_{1}intervention+\beta_{2}social\;support+\beta_{3}{share\;food+\beta_{4}eat\;seasonally+\beta_{5}try\;new\;food+\beta_{6}self\;efficacy+\varepsilon }^{6}$$

## Results

Baseline characteristics of the participants in the randomized trial are reported elsewhere [8]. Participants in the overall trial, on average, were 41 y of age, 82% were female, 34% were Hispanic, 65% were college graduates, and 31% had <1 y of experience with gardening (data not shown). Qualitative analysis results are presented first followed by the quantitative analysis results.

### Qualitative analysis

Baseline characteristics of the qualitative interview participants subsample are displayed in Supplemental Table. Seventy-one percent of the qualitative study participants were female, their mean age was 41 y, 21% were Hispanic, 59% were college graduates, and 49% had less than 1 y of experience with gardening.

The majority of the qualitative study participants (n = 27) described their diets as healthy, valued eating healthy foods, or had recently changed their diet to be healthier in the year or years before they began gardening. Even so, participants in the qualitative interviews reported dietary changes as a result of their participation in gardening that fell into specific categories (some participants fell into more than one of the categories). About 50% of the qualitative study participants reported eating more fruits and/or vegetables because of their garden, 8 participants reported increasing the amount of produce in their diet throughout the year, and 6 participants reported that they ate more vegetables but only during the summer gardening season. Most participants felt that the garden helped sustain their already healthy diet and/or they substituted vegetables they normally ate with vegetables from the garden (n = 27). Finally, 7 participants said that the garden helped improve their overall diet quality, including eating fewer processed and convenience foods. Only 2 participants reported that their diet did not change in any way due to gardening. One of these participants produced very little food from their garden (enough for a few meals) and the other had some garden produce stolen and donated most of the rest.

### Reasons for diet changes

Participants who experienced a dietary change due to their participation in the community garden were asked why they believed that these changes occurred. Responses are described below.

#### Availability of garden produce

All qualitative study participants successfully grew fresh food in their gardens and took some home to eat. The amount of garden produce harvested and consumed ranged from having only enough for a few meals (*n* = 5) to having so much food they needed to give some of it away (*n* = 17). Most participants articulated that simply having garden produce available motivated them to eat it. Harvesting the garden produce was convenient. As one participant said, “I would eat healthier because of all the things… in the garden” (P.H). For some participants, becoming accustomed to having and eating garden produce encouraged them to seek out vegetables in the winter, which changed their diet year round. One participant (P.LL) appreciated the convenience of having fresh food continuously available in the garden ready for picking when needed (in other words, storing it in the garden rather than their refrigerator), which meant that the produce was less likely to spoil.

#### Caretaking and valuing garden plants

About half of the participants (*n* = 13) felt connected to the food they produced in the garden after spending time caring for plants throughout the growing season. One participant described how food from their garden is something “you’ve earned” because you see it “from start to finish” (P.V). Another participant described how tending to garden plants and spending time gardening throughout the season made him feel more invested in his food. He said, “When you grow your own food, you’re invested in it, so might as well eat it, might as well make the salads instead of doing the hot dogs” (P.Q). One participant described the garden as an environment she could tailor to her preferences, meaning that she could grow things she liked and wanted to eat. She said, “Everything I grow is a part of me, is something that I put in there, is something that I wanted not anybody else” (P.GG).

Several participants (*n* = 9) noted that their diet was influenced by not wanting to waste food they grew themselves. They described how their investment of time, effort, and care in growing produce contributed to placing increased value on garden produce and feeling a greater connection to the food they harvested from their gardens. One participant said that their plants “almost become like part of your family” (P.Y). Another participant explained, “We waste less when we are connected to our food in one way, shape or form beyond plastic wrap in a grocery store. There’s a sense of respect for the food you don’t get by walking up and down an aisle… Respecting the food, respecting the people that took time to grow it… I don’t want to waste that effort” (P.D).

#### Pride, accomplishment, and self-reliance

The majority of the participants (*n* = 20) associated consumption of their garden produce with a sense of pride for their work in the garden and a feeling of accomplishment when they harvested their produce. One participant (P.H) described the excitement and pride of growing garden produce: “It was very exciting when I got to see my things growing…it was really rewarding when you see your things that you put a lot of work on them.” Several participants discussed how harvesting and eating garden produce after all the work they had put in throughout the season to have viable plants was “satisfying.” Other participants noted that producing their own food in the garden contributed to feelings of self-reliance. These participants took pride in their ability to produce food for themselves and their families. This in turn caused them to value garden produce and value eating the foods that they had worked hard to grow. As one participant said, “[it was] amazing to see the stuff I grew there…. [I felt like a] strong-willed badass... this is something I planted, something I grew. So it empowered me” (P.DD).

#### Aesthetic connection to nature and natural food

Most participants (*n* = 25) described finding garden produce more enjoyable, higher quality, better tasting, and/or more aesthetically pleasing than that available in supermarkets and grocery stores. For some participants, they connected the perceived superior quality of garden produce as a result of their efforts in growing it. For other participants, the high quality of garden produce was a result of their knowledge of the gardening practices in the community garden, believing organic or local foods to be of higher quality. For others, this was related to the aesthetic experience of being in the garden. As one participant said, “It feels like I’m getting back to my roots like how people back in the day were growing out their own food and just getting back to that route. Feeling the earth, growing your own food, seeing your own vegetables grow, and eating them, it’s so rewarding” (P.P).

#### Self-efficacy for healthy diet: trying new foods and cooking

Most participants (*n* = 25) described an increase in self-efficacy of incorporating fruits and vegetables into their diet, including trying new foods, gaining new cooking or food preparation skills, or cooking more at home due to their participation in the community garden. Although most participants grew foods familiar to them, a few participants described growing new foods or trying new foods grown by another gardener. Similarly, several participants described finding cooking and eating inspiration from other gardeners’ plots and from the garden environment in general. For example, 1 participant sought out different foods at grocery stores after seeing what another gardener was growing in their plot, prompting them to add variety to their diet. A few participants gained cooking and food preparation skills by learning to cook new foods, learned recipes from other gardeners, or learned to cook or preserve food for the first time due to the high availability of garden produce during the gardening season. As 1 participant (P.D), said “If it hadn’t been for the community garden, I don’t think I would be cooking.” Another participant described how the garden and garden produce promoted her to be more creative with her eating and cooking: “[The garden] just helped me be more creative. Gardening just adds to the love of eating and being creative…” (P.J). Conversely, 2 participants described struggling with cooking or preserving garden produce, which resulted in them eating less garden produce than they would have liked (P.O and P.MM).

Some participants described general improvements in their self-efficacy for having a healthy diet due to their involvement in the community garden. “I think for me, it displays an opportunity to create your own health benefits, I guess because, because you’re doing your own food, and you’re not doing any of the processed foods” (P.Y). Similarly, another participant described how the garden improved her health through the nutritious garden produce: “it helps my health so I loved it… [because it] provides nutrients” (P.J).

#### Eating seasonally and appreciation of fresh, sustainable, organic, and/or local food

Most participants (*n* = 24) described that the garden changed their feelings about eating seasonally and described the pleasure this brought them. Although a few participants had this value before the study and the garden reinforced these existing values, eating seasonally was a new experience for most qualitative study participants. Some participants made connections beyond their own garden produce to appreciate home-grown, sustainable, organic, and/or local (and mis-shaped) foods in general. One participant said, “I feel like I’m making an impact environmentally, too. It’s really small, but it’s not having to buy stuff from the store. It’s just a nice activity that’s healthy, environmentally friendly and relaxing” (P.II).

#### Sharing garden food with others

Most of the participants (*n* = 25) shared food from their gardens with their friends and family, enjoyed eating the garden food together with others, and/or donated their produce to local organizations. Several participants mentioned that this was an important feature of their experience and enhanced their feelings of pride in the garden. One participant who explained that he shared his tomatoes and cucumbers with friends said, “I was popular for that” (P.Y). Another participant said, “that’s one reason I like it because it helps others” (P.U). Another explained that her husband was excited by the food she would bring home: “He was like, ‘wow, I didn’t even know these things existed….’”. She went on to say, “The joy you can bring to people for bringing something that is good for them, like for my family…see that they enjoy and they really care, and that you benefit from there, it’s very rewarding. Maybe the highlight of the whole thing” (P.H).

Several participants brought children to the garden with them, and a particular highlight of gardening was seeing the garden’s impact on them. One participant described her child as eating tomatoes “like apples… they were so plentiful that she would just pick it up and eat it. That was new” (P.L). Another participant said that her child said, “Mom, Dad, this tastes better. I’m like, ‘yes that’s because we did it’” (P.S).

Not only did participants share food with others, but many received food and/or recipes from other gardeners. A few participants described their appreciation for learning about new foods from gardeners from other cultures.

#### Financial benefit

Eight participants noted a financial benefit to gardening and were motivated to garden by these cost savings. One participant explained that before gardening, her family could afford only canned or frozen vegetables, but now they were eating their own fresh vegetables and also seeking out fresh vegetables from the grocery store. As she explained, “[the garden] changed our diet. We had all these vegetables and as much as we wanted,” (P.S). Another participant who described preserving her garden food said she was eating higher quality food during the garden season and throughout the year, and that she had saved money at the grocery store and on gifts (P.I). One participant’s children made this financial connection as well. She explained that they said, “Wow, this is the stuff we usually buy at the store and now we can just go to our own garden” (P.L).

### Quantitative results

Of the 281 participants enrolled in the CAPS trial, 243 participants met the inclusion criteria for this post hoc subanalysis (Figure). Mediator variables to be assessed were selected based on the qualitative results. Table 1 displays the outcome and mediator variables at baseline (T1). There were statistically significant differences at baseline for the mediator variables social support for fruit and vegetable intake and sharing food but not the other outcome or mediator variables.

**FIGURE:**
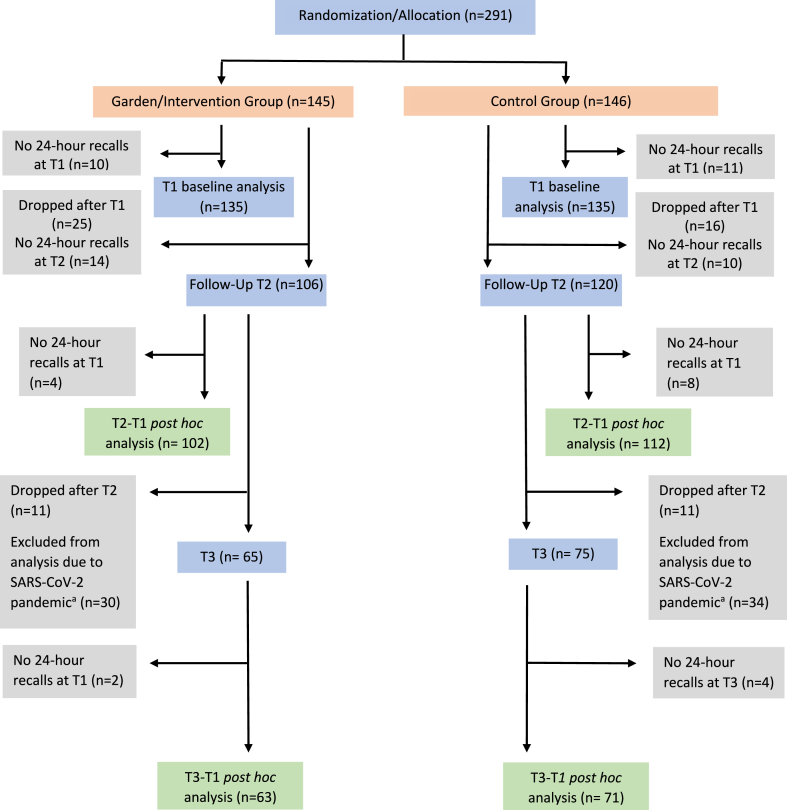
CONSORT diagram. Because the SARS-CoV-2 pandemic affected data collection and might have had an impact on health behaviors, the study team agreed to exclude Wave 3, T3 data from the analysis. The decision was made a priori, while the blind was still in force.

**TABLE 1:** Outcome and mediator variables at baseline (T1)

Outcome	Control *n* = 135		Intervention *n* = 135		Total *n* = 270		Statistical test for difference
	Mean	SD	Mean	SD	Mean	SD	
Combined fruit and vegetable intake (servings/d)	4.94	2.81	4.91	2.83	4.92	2.81	*P* value = 0.92
Fruit intake (servings/d)	1.22	1.19	1.33	1.28	1.28	1.23	*P* value = 0.44
Vegetable intake (servings/d)	3.73	2.25	3.58	2.42	3.64	2.33	*P* value = 0.61
Garden vegetable intake (servings/d)	3.12	2.06	2.99	2.05	3.04	2.05	*P* value = 0.60
Social support for fruit and vegetable intake (scale score: −2 to 2)	0.17	1.02	0.21	0.98	0.19	1.00	*P* value = 0.01
Sharing food (scale score: 0–4)	2.05	0.71	2.21	0.68	2.13	0.70	*P* value < 0.001
Eating seasonally (% who eat seasonally)	0.56	0.50	0.50	0.50	0.53	0.50	*t* = −0.82,
							df = 764.47,
							*P* value = 0.41
Self-efficacy to eat fruit and vegetables (scale score: −2 to 2)	0.92	0.70	0.90	0.74	0.91	0.71	*P* value = 0.85
Vegetable preference (scale score: −40 to 40)	21.66	8.69	21.95	8.12	21.80	8.40	*P* value = 0.48

Table 2 reports differences in dietary intake between the control and intervention groups from T1 to T2 and from T1 to T3 for combined fruit and vegetables, fruit only, vegetables only, and garden vegetables. Participants in the intervention group increased their servings of vegetables by 0.63 servings of vegetables (*P* = 0.047) and 0.67 servings of garden vegetables (*P* = 0.015) more than the control group from T1 to T2. There were no significant differences between the control and intervention groups with regard to changes in combined fruit and vegetable and fruit intake from T1 to T2. There were also no significant changes in dietary intake from T1 to T3 for any of the outcomes.

**TABLE 3:** Mediation model results

Direct effects (*n* = 214)					
	Estimate	se	Standardized estimate	95% CI	*P* value
Social support for fruit and vegetable intake⇒ garden vegetable intake	0.161	0.229	0.057	[−0.284, 0.614]	0.48
Sharing food with others ⇒ garden vegetable intake	−0.069	0.259	−0.020	[−0.590, 0.439]	0.79
Eating seasonally ⇒ garden vegetable intake	0.539	0.286	0.157	[−0.017, 1.101]	0.06
Self-efficacy to eat fruits and vegetables ⇒ garden vegetable intake	0.095	0.249	0.031	[−0.362, 0.631]	0.70
Vegetable preference ⇒ garden vegetable intake	0.011	0.027	0.030	[−0.044, 0.062]	0.70
Intervention (community gardening) ⇒ garden vegetable intake	0.558	0.275	0.138	[0.025, 1.101]	0.04
Intervention (community gardening) ⇒ social support for fruit and vegetable intake	0.030	0.094	0.021	[−0.158, 0.213]	0.75
Intervention (community gardening) ⇒ sharing food	0.026	0.076	0.022	[−0.120, 0.174]	0.72
Intervention (community gardening) ⇒ eating seasonally	0.172	0.073	0.147	[0.021, 0.306]	0.02
Intervention (community gardening) ⇒ self-efficacy to eat fruits and vegetables	0.107	0.084	0.083	[−0.061, 0.271]	0.20
Intervention (community gardening) ⇒ vegetable preference	1.314	0.810	0.113	[−0.355, 2.834]	0.10
Social support for fruit and vegetable intake with sharing food	0.006	0.028	0.014	[−0.051, 0.060]	0.83
Social support for fruit and vegetable intake with eating seasonally	0.015	0.028	0.037	[−0.039, 0.071]	0.58
Social support for fruit and vegetable intake with self-efficacy to eat fruits and vegetables	0.085	0.032	0.186	[0.028, 0.151]	0.007
Social support for fruit and vegetable intake with vegetable preference	−0.126	0.346	−0.031	[−0.844, 0.512]	0.72
Sharing food with eating seasonally	0.028	0.019	0.083	[−0.007, 0.066]	0.13
Sharing food with self-efficacy to eat fruits and vegetables	0.091	0.027	0.240	[0.043, 0.149]	0.001
Sharing food with vegetable preference	0.624	0.295	0.183	[0.105, 1.263]	0.03
Eating seasonally with self-efficacy to eat fruits and vegetables	0.033	0.023	0.088	[−0.010, 0.078]	0.15
Eating seasonally with vegetable preference	0.333	0.263	0.100	[−0.154, 0.869]	0.21
Self-efficacy to eat fruits and vegetables with vegetable preference	0.291	0.227	0.079	[−0.168, 0.738]	0.20
Intercept (garden vegetable intake)	−0.354	0.196	−0.176	[−0.759, 0.009]	0.07
Intercept (social support for fruit and vegetable intake)	0.002	0.062	0.003	[−0.12, 0.124]	0.97
Intercept (sharing food)	−0.004	0.049	−0.006	[−0.098, 0.093]	0.94
Intercept (eating seasonally)	−0.032	0.050	−0.055	[−0.127, 0.069]	0.52
Intercept (self-efficacy to eat fruits and vegetables)	0.034	0.056	0.052	[−0.075, 0.146]	0.55
Intercept (vegetable preference)	−0.399	0.480	−0.069	[−1.347, 0.514]	0.41
Residual (garden vegetable intake)	3.825	0.498	0.940	[3.033, 5.123]	<0.0001
Residual (social support for fruit and vegetable intake)	0.506	0.045	1.000	[0.428, 0.608]	<0.0001
Residual (sharing food)	0.351	0.036	1.000	[0.288, 0.429]	<0.0001
Residual (eating seasonally)	0.337	0.030	0.979	[0.283, 0.400]	<0.0001
Residual (self-efficacy to eat fruits and vegetables)	0.413	0.052	0.993	[0.322, 0.531]	<0.0001
Residual (vegetable preference)	33.100	4.583	0.987	[25.492, 43.604]	<0.0001
**Indirect effects (*n* = 214)**					
	Estimate	se	Standardized Estimate	Bootstrap 95% CI	
Total indirect effect	0.120	0.089	0.030	[−0.019, 0.338]	
Intervention (community gardening) ⇒ social support for fruit and vegetable intake ⇒ garden vegetable intake	0.005	0.028	0.001	[−0.028, 0.101]	
Intervention (community gardening) ⇒ sharing food ⇒ garden vegetable intake	−0.002	0.022	0.000	[−0.074, 0.029]	
Intervention ⇒ eating seasonally ⇒ garden vegetable intake	0.093	0.067	0.023	[0.002, 0.284]	
Intervention (community gardening) ⇒ self-efficacy to eat fruits and vegetables ⇒ garden vegetable intake	0.010	0.035	0.003	[−0.034, 0.124]	
Intervention (community gardening) ⇒ vegetable preference ⇒ garden vegetable intake	0.014	0.043	0.003	[−0.051, 0.136]	

Table 3 displays the results of the mediation analysis, which was performed to assess the mediating roles of social support for fruit and vegetable intake, sharing food, eating seasonally, self-efficacy to eat fruits and vegetables, and vegetable preferences in the relationship between community gardening and garden vegetable intake. Because the model was a saturated model, we did not report model fit indexes. Community gardening was positively associated with the intake of garden vegetables (***β*** = 0.558, *P* = 0.04), and among the potential mediators, community gardening was also positively associated with eating seasonally (***β*** = 0.172, *P* = 0.02). The indirect effect of the gardening intervention through eating seasonally was significant based on the bootstrapping CI [0.002, 0.284]. None of the other mediators were significantly associated with gardening or with fruit and vegetable intake.

**TABLE 2:** Difference score analysis for fruit and vegetable intake

**Outcome**	T2 – T1 (*n* = 214)					T3 – T1 (*n* = 134)				
	*β* Coef.	SE	*R*^2^	Adjusted *R*^2^	*P* value	*β* Coef.	SE	*R*^2^	Adjusted *R*^2^	*P* value
Combined fruits and vegetables	0.68^1^	0.36	0.016	0.011	0.07	0.83^1^	0.47	0.023	0.015	0.08
Fruit	0.05^1^	0.19	0.000	−0.004	0.79	0.34^2^	0.23	N/A	N/A	0.15
Vegetables	0.63^1^	0.31	0.019	0.014	0.047	0.49^1^	0.40	0.011	0.004	0.22
Garden vegetables	0.67^2^	0.27	N/A	N/A	0.015	0.36^2^	0.36	N/A	N/A	0.32

1 Single level regression model.

2 Multilevel regression model.

## Discussion

In this study, the first RCT of community gardening, we found that from baseline to harvest, participants randomized to community gardening significantly increased their intake of vegetables and garden vegetables, but not their combined intakes of fruits and vegetables or fruits alone compared with nongardening control participants. We did not find any statistically significant differences between the gardening and control groups from baseline to winter follow-up. Furthermore, gardening significantly increased participants’ seasonal consumption of fruits and vegetables at harvest. Several RCTs have been conducted on school gardens and they were found to improve dietary intake among children; for example, the Texas Sprout found that school gardening, cooking, and nutrition intervention increased vegetable intake among 3rd to 5th grade students [16]. Only 3 previous studies of gardening and diet among adults are randomized trials and all assessed home gardening. In a study among older cancer survivors (age 60+), Demark-Wahnefried et al. [17] found that participants who received support for planting home gardens over a 1-y period increased their combined fruit and vegetable intake by about 1 serving per day compared to controls who showed no significant change in fruit and vegetable intake. Similarly, a study of breast cancer survivors found an increase of 0.86 serving of vegetables per day in the intervention group, compared with a 0.23 serving increase in the control group [18]. Blakstad et al. found that gardening improved dietary quality in Tanzanian women who received garden seeds, nutrition education, and gardening instruction. Women in the intervention group consumed 0.5 more food groups per day than those in the control group, and were 14% more likely to consume ≥5 food groups (of 10 possible food groups) per day than women in the control group [19]. Our study, along with these other RCTs, provides evidence to support gardening as an important public health nutrition intervention.

Our qualitative results mirrored our quantitative findings. About half of the qualitative study participants reported increased vegetable intake due to gardening, with many of these participants reporting increased vegetable intake only during the harvest months. A small subset of qualitative study participants reported that gardening helped them improve the overall quality of their diet by reducing the intake of fast foods and highly processed foods. Finally, some qualitative study participants reported that they did not change their overall diet, but instead ate vegetables from the garden instead of vegetables they would have eaten anyway. This diet outcome, eating vegetables from their garden, would not be discernable with a pre- and postquantitative diet assessment. Yet, many gardeners in our study and others reported a psychological and even spiritual improvement in their lives that went along with the act of eating fresh garden produce that one has grown themselves [5,[20], [21], [22]]. Furthermore, the act of growing food instilled in participants a greater appreciation for food quality, how food is grown, and food preparation.

We explored the reasons for increased intake of garden vegetables through both qualitative interviews and quantitative mediation analysis. We observed that the process of gardening engagement led to 5 within-garden outcomes, which, in turn, led to beyond-garden diet changes. The first, growing and having available fresh and delicious food, was an environmental change. A few studies have measured the amount of produce grown by gardeners. For example, one study conducted in San Jose, California, found that community garden practices are similar to biointensive high-production farming and can produce 0.75 lb vegetables per square foot of growing space [23].

Three within-garden outcomes described emotional processes: an emotional attachment to garden plants leading to a sense of personal responsibility for the plants, which resulted in nurturing the plants, an enhanced aesthetic connection with nature and garden produce, and feelings of accomplishment and pride for their work in the garden and for the produce they harvested. In other studies, gardeners overwhelmingly report that the food they grow in their gardens tastes better than the produce they could purchase at the grocery store [[24], [25], [26]]. Gardeners attribute this improved taste to freshness, the emotional attachment they have to food they have grown themselves, and the pride and satisfaction derived from the processes involved in growing food and seeing your activities (literally) bear fruit [25,27,28]. Gardeners often value organically grown produce because it is conceived as healthier and many gardeners expressed comfort over having control and knowledge of how the produce from their gardens was grown [27,[29], [30], [31]]. Many, but not all, gardening participants interviewed experienced socializing in the garden, which led to sharing food with others, trying new foods, and exchanging recipes. Sharing food and trying new foods are a common theme among community gardening and allotment studies [5,21,32,33].

Some qualitative interview participants reported saving money on food. Surveys of gardeners and qualitative studies have found that gardeners perceive that they save money due to gardening, but the results from the studies on gardening and cost savings are mixed [5,23,29,[34], [35], [36], [37]]. Over 50% of home gardeners reported saving more than $480 annually on food from gardening in 1 study conducted in a low-income area of San Jose, California, with 25% reporting savings of over $720 [35]. Many programs exist nationwide that provide these inputs for free or at a low cost to both home and community gardeners. For example, DUG, the partner organization for this research implements a Grow a Garden program, which provides seeds and transplants for low-income gardeners.

Using the findings from the qualitative interviews as a guide, we conducted a post hoc analysis of mediators using the quantitative data. We found that participation in a community garden significantly increased particippants' season eating and that seasonal eating was associated with a greater intake of garden vegetables. Other mediators including social support to eat fruit and vegetables, sharing food with others, and self-efficacy to eat fruit and vegetables were neither significantly changed by community gardening nor associated with garden vegetable intake. There have been few quantitative studies assessing the mediators between gardening and diet. Veen et al. found that participants who were motivated to garden by vegetable production ate a larger percentage of vegetables from the garden [38]. In a cross-sectional study, neighborhood aesthetics, social involvement, and community garden participation were significantly associated with fruit and vegetable intake [39]. Further research should assess other mediators found through qualitative studies such as our study in quantitative mediation studies, such emotional connection to garden produce through caretaking and sense of accomplishment, improved taste and aesthetics of garden produce, sharing food with others, and cooking and trying new foods.

One limitation of the study was the exclusion of data from Wave 3, T3 due to the COVID-19 pandemic, which may have resulted in lower statistical power to detect differences. In addition, the qualitative study participants were a subset of the trial participants and may not be representative of the entire sample.

Our study assessed the dietary impact of real-world community gardening conditions; no other means for influencing the diet, such as nutrition education, were provided to the participants. The results of our study demonstrate that community gardening is an effective socio–environmental public health intervention that acts through multiple pathways to improve diets. Our post hoc analyses of the CAPS trial found that participating in a community garden increased vegetable intake and seasonal eating. Qualitatively, gardeners explained that their diet changes occurred because of the availability of garden produce, the emotional connections they felt with their plants and gardens, sense of pride and accomplishment in growing their own food and not wanting to waste it, the improved taste of the garden produce, and trying new foods and recipes.

In summary, our study found that community gardening was effective at improving diets for people from all walks of life, including those who live in both higher- and lower- resourced areas. Thus, gardening, as a nature-based intervention, can be a vital pathway for the prevention of both chronic and infectious disease outcomes. Community gardening is a viable means of increasing the amount and quality of vegetables consumed among participants and should be recognized as an important venue for improving the nutritional health of communities.

## Funding

This study was supported by a Research Scholars Health Equity Grant from the American Cancer Society (University of Colorado Boulder, 130091-RSG-16-169-01-CPPB; Michigan State University; and University of South Carolina Subawards); the University of Colorado Boulder; the University of Colorado Cancer Center; USDA National Institute of Food and Agriculture, Michigan AgBioResearch Hatch projects (MICL02410, MICL02711); and Wayne State University Department of Nutrition and Food Science Faculty Start-up Fund. The funders of the study did not participate in any way in the study design; data collection, analysis, and interpretation; or writing of the manuscript.

## Author contributions

The authors’ responsibilities were as follows – KA: co-conceptualized the study and supervised the analyses and writing; KA, TGH, JRH: designed the research; JRH, TGH: managed dietary data collection; AWB, EC, KL: collected the data; KA, AWB, EC, KL, WM: analyzed the data; KA, EC, AWB, KL, WM, JRH: wrote the paper, contributed to drafting the manuscript, provided final approval to publish, and are accountable for all aspects of the manuscript; all authors: contributed to the interpretation of results, revised the manuscript, provided relevant intellectual input, had full access to all the data in the manuscript, approved the decision to submit for publication, and read and approved the final manuscript.

## Conflict of interest

JRH owns a controlling interest in Connecting Health Innovations LLC (CHI), a company that has licensed the right to his invention of the dietary inflammatory index (DII) from the University of South Carolina to develop computer and smart phone applications for patient counseling and dietary intervention in clinical settings. JRH confirms that the subject matter of this manuscript will neither have any direct bearing on that work nor have that activity have any influence on this project. All other authors report no conflicts of interests.

## Data availability

The deidentified participant data, data dictionary, the statistical code, and the qualitative data and codebook may be shared upon request pending approval by the trial investigators and relevant institutional review boards.

## Appendix A Supplementary data

Multimedia component1 Multimedia component1
