# Supplementary material for: Community Gardening Increases Vegetable Intake and Seasonal Eating From Baseline to Harvest: Results from a Mixed Methods Randomized Controlled Trial
Source: Curr Dev Nutr. 2023 Apr 15;7(5):100077. doi: 10.1016/j.cdnut.2023.100077 (PMC10196338; doi:10.1016/j.cdnut.2023.100077)
Supplement: Multimedia component1 [file mmc1.docx]

**Supplemental Table 1: Demographic Characteristics of the Qualitative Interview Participants**

| **Demographic Characteristic** | **N=34** |
| --- | --- |
| Age (mean (range)) | 41 (20-70) |
| Gender (n (%))  Female  Male | 24 (71%)  10 (29%) |
| Ethnicity (n (%))  Non-Hispanic  Hispanic | 27 (79%)  7 (21%) |
| Education (n (%))  College 4 years or more (college graduate)  College 1-3 years (some college or technical school)  Grade 12 or GED (high school graduate)  Grades 9-11 (some high school)  Grades 1-8 (elementary) | 20 (59%)  10 (29%)  2 (6%)  1 (3%)  1 (3%) |
| Number of Children in Household (mean (range)) | 0.65 (0-3) |
| Food Security (n (%))  Food secure  Food insecure | 25 (74%)  9 (26%) |
| Previous Gardening Experience (n (%))  None  <1  1-2 years  3-5 years  5-10 years  10+ years | 8 (24%)  12 (25%)  6 (18%)  4 (12%)  2 (6%)  2 (6%) |
